# A Case Study on Pyogenic Granuloma With Review of the Literature: An Unexpected Sequela or a Complication of Dental Extraction?

**DOI:** 10.7759/cureus.46592

**Published:** 2023-10-06

**Authors:** Ketan A Dodal, Rozina Vishnani, Amit Reche, Rahul R Bhowate, Rajanikanth K

**Affiliations:** 1 Public Health Dentistry, Sharad Pawar Dental College, Datta Meghe Institute of Higher Education and Research, Wardha, IND; 2 Oral and Maxillofacial Surgery, Sharad Pawar Dental College, Datta Meghe Institute of Higher Education and Research, Wardha, IND; 3 Oral Medicine and Radiology, Sharad Pawar Dental College, Datta Meghe Institute of Higher Education and Research, Wardha, IND

**Keywords:** non-neoplastic lesions, benign vascular tumor, extraction, soft tissue growth, pyogenic granuloma

## Abstract

Pyogenic granuloma is a common reactive oral lesion primarily found in the gingiva and rarely in extraction sockets. While it can develop at any age, it is more prevalent in the third and fourth decades of life with a higher occurrence in females. Various factors contribute to its development and surgical removal is the gold standard treatment; however, there are various other methods available. This case report documents a rare event in which a female patient in her early 40s presented with an exophytic lesion affecting the extraction socket of her maxillary right lateral incisor. The lesion was effectively removed through surgical excision. Additionally, it explores the clinical features and pathogenesis of this lesion. The purpose of this case report is to shed light on the uncommon incidence of pyogenic granuloma following tooth extraction. This non-neoplastic vascular growth often presents as an erythematous, ulcerated lesion with a tendency to bleed, with either a sessile or pedunculated base. Our case is one of only five instances documented in the literature, underscoring the importance of knowledge and timely response in such unusual circumstances. We emphasize the significance of early detection and management for improved patient outcomes and a better understanding of this rare condition.

## Introduction

One of the most frequent pedunculated or sessile lesions in the oral cavity is the pyogenic granuloma, an inflammatory hyperplasia of vascular origin. Secondary triggers like trauma, local irritation, and hormonal imbalance cause pyogenic granuloma [1]. The lesion typically ranges between 0.5 cm and 2 cm, but it can grow alarmingly quickly, reaching that size in four to seven days. The first case to be notified of pyogenic granuloma was in 1897 by Poncet and Dor, and in that period, it was known as botryomycosis hominis. The term was coined by Hartzell in 1904; however, after these years of cytological advancement, Angelopoulos AP stated that on looking out for its hypocellular structure, it should be termed a hemangiomatous granuloma [2]. Pyogenic granuloma is mostly encountered in the maxilla than the mandible; it has a higher affinity for the gingiva, followed by the palate and buccal mucosa. Only five cases have been documented, including this one, which showed the emergence of the extraction socket as sequelae or a complication of extraction. Our case stands out because it occurred in the anterior maxilla, an uncommon location. The posterior maxilla and posterior mandible were the sites where the lesion was encountered in the other four occurrences [3,4].

## Case presentation

A 40-year-old female patient reported to the Department of Oral and Maxillofacial Surgery with a chief complaint of swelling in the upper anterior region of the jaw, persisting for five months. The onset of the lesion was noted approximately five months ago, initially manifesting as a small growth that gradually increased in size up to a centimeter. Importantly, the lesion did not elicit pain, bleeding, or discharge. The patient's dental history revealed that she had deep occlusal caries for which she underwent an extraction of the maxillary right lateral incisor six months back and the maxillary central incisor three years ago. Furthermore, she had a medical history of undergoing a resection procedure for a uterine myoma 10 years back, and in the literature, there is no evidence of documentation of the association between pyogenic granuloma and uterine myoma. There was no history of systemic illness like diabetes or hypertension. Notably, there was no habit history of tobacco, alcohol consumption, or smoking.

**Figure 1:**
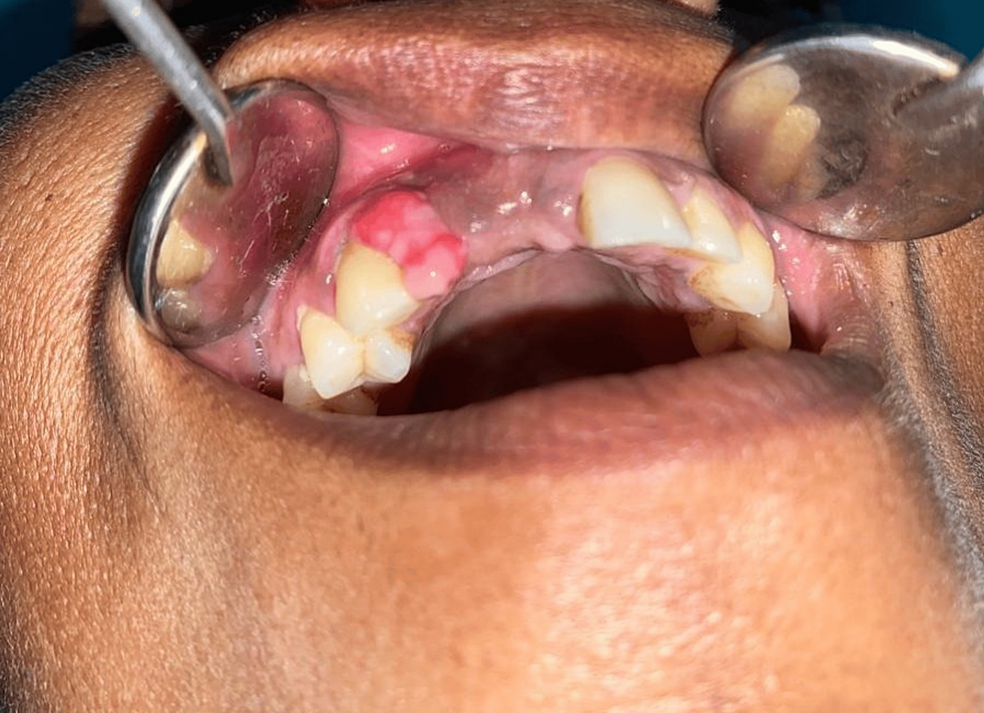
Clinical presentation of the presented case. The presented lesion is featured with an ulcerated surface with distributed areas of erythema, which shows a tendency to bleed (no bleeding was encountered during examination). The size of the lesion measured was 1 x 0.75 centimeters.

**Figure 2:**
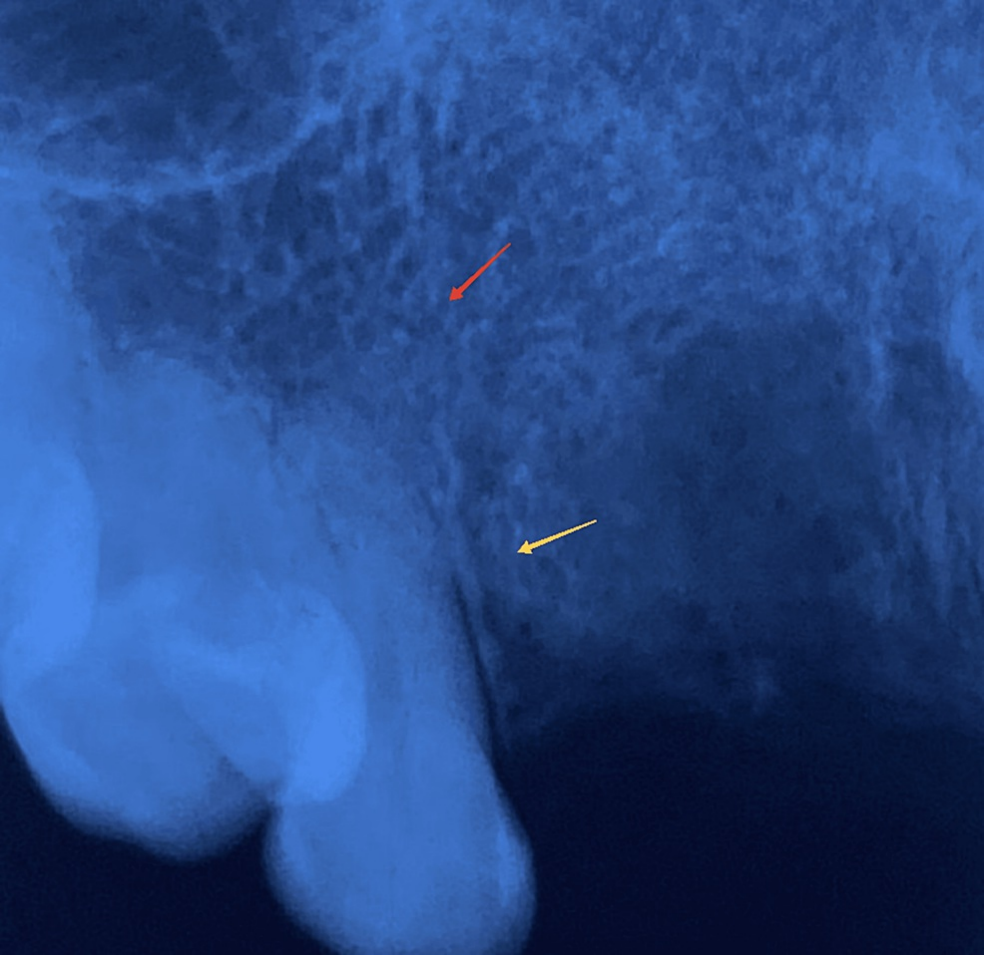
Intraoral periapical radiograph of the lesion. The red arrow depicts the trabecular condensation and the yellow arrow depicts apical periodontal space widening.

On examination, a projecting soft tissue growth above the socket of the maxillary right lateral incisor was discovered. The dimensions of the growth were measured as approximately 1 x 0.75 centimeters. The lesion was roughly oval with a raised and smooth surface, displaying partial erythematous coloring and well-defined margins. Upon palpation, the lesion was found soft, non-tender, and attached by a pedunculated base (Figure 1) and no bleeding from the lesion was noted. An intraoral periapical radiograph was taken, which showed dense trabecular condensation in the vicinity of tooth 12, along with widened apical periodontal space associated with the right maxillary canine (Figure 2).

Considering the amalgamated clinical and radiographic findings and differential diagnosis, which included gingival fibroma and peripheral ossifying fibroma, a provisional diagnosis of pyogenic granuloma was established.

**Figure 3:**
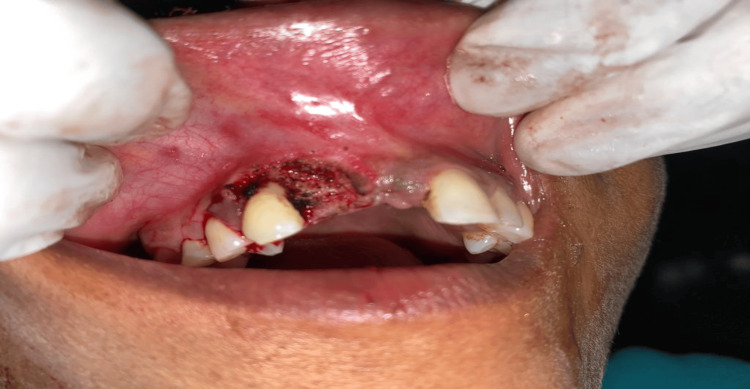
Surgical site with achieved hemostasis.

**Figure 4:**
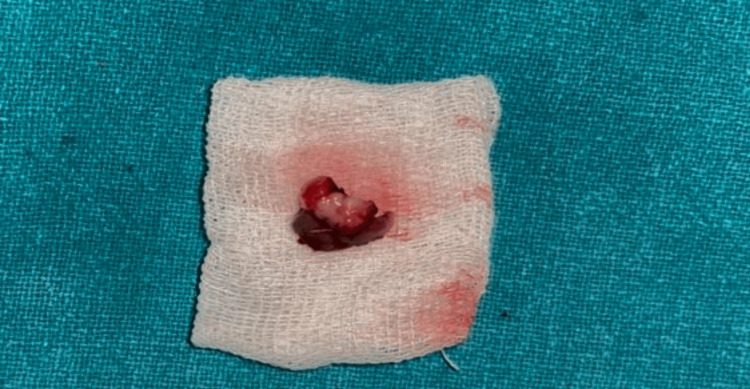
Excised lesion.

The case was planned for surgical excision under local anesthesia. Prior to surgery, the patient underwent a general examination and systemic examination and had all necessary blood tests, which were all within normal parameters. Following all aseptic protocols, under local anesthesia (2% lidocaine with 1/100,000 epinephrine), the lesion was marked 1 mm away from its margin. The surgical excision was done using blade number 15. The surgical excision was mostly focused on excising the lesion from its base and including removal of the periosteal layer of bone. After excising the complete lesion, the bleeding points were cauterized, and a thorough wash with normal saline was given. The surgical site was examined and evaluated, and hemostasis was achieved (Figures 3, 4). Pressure packs were placed, and the patient was discharged with postoperative instructions and recalled after seven days for follow-up. During this time, the patient was kept under antibiotic and analgesic coverage. The excised tissue was sent to the Department of Oral Pathology for histopathological study. The present case was followed up for six months with no recurrence.

**Figure 5:**
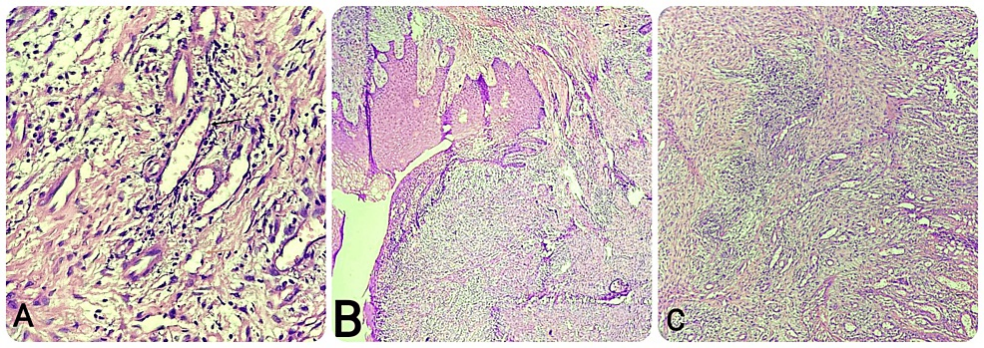
Histological presentation of the hematoxylin and eosin-stained soft tissue under the microscope. (A) Under 40x view, which shows blood vessels marked with an orange arrow and vascular cleft marked with a black arrow. (B) 4x view under the microscope shows the parakeratinized stratified squamous epithelium lining the lesion. (C) 10x view shows the collagen fibers embedded into the connective tissue of the stroma of pyogenic granuloma.

The histopathological examination of the excised soft tissue stained with hematoxylin and eosin showed a para-keratinized stratified squamous epithelium exhibiting an underlying granulation tissue. The underlying connective tissue exhibits characteristics such as extensive vascular clefts and concentrated clusters of fibroblasts and endothelial cells, which are indicative of pyogenic granuloma (Figure 5). The final diagnosis based on histopathological examination was pyogenic granuloma.

## Discussion

In the oral cavity, pyogenic granuloma is considered a non-neoplastic growth, but it still demands significant attention in terms of diagnosis, prevention, management, and treatment. This is because pyogenic granuloma can manifest in atypical ways, sharing characteristics with different conditions, making its clinical diagnosis quite challenging. To ensure accurate identification and minimize the chances of misdiagnosis, it is crucial to perform biopsies on all suspected pyogenic granuloma cases. When considering a differential diagnosis, it is essential to include peripheral ossifying fibroma and gingival fibroma as potential conditions with some similar presentations, which need to be examined. To rule out pyogenic granuloma, it is necessary to first understand the characteristic features of pyogenic granuloma, which can be categorized based on its occurrence, clinical presentation, radiological features, and histological features. Pyogenic granuloma is typically observed in individuals aged between 30 and 50 years. The epidemiological trends for pyogenic granuloma are different in different studies. According to one review, there is a slight predominance of males during their second decade and female predominance during the third and fourth decades. One study suggests that the occurrence of pyogenic granuloma is more common in females than males irrelevant of their age group.

The characteristic clinical appearance of a pyogenic granuloma entails a minute deep red to reddish-purple lesion that can manifest either as a sessile or pedunculated growth on the gingiva. The lesion surface may exhibit varying textures ranging from smooth to lobular, occasionally presenting wart-like attributes. Clinically, this condition presents with lesions that appear erythematous and have a tendency to bleed easily. These lesions can manifest as either sessile or pedunculated growths [3]. While radiological features are rarely observed, there have been isolated cases such as one documented by Ketelin Juliane Dal Pra et al. [5], in which radiographs showed bone loss, particularly horizontal bone loss. Histologically, pyogenic granuloma is characterized by a significant infiltration of lymphocytes, plasma cells, and neutrophils within arterial regions demarcated by endothelial lining. According to Cawson et al.'s [6] analysis of the lesion's vascular supply and rate of proliferation, two histologic variants were identified, which are lobular capillary hemangioma (LCH) and non-lobular capillary hemangioma (NLCH). Increased vessels organized in lobular architecture are found in the histologic presentation of LCH, and the NLCH type exhibits strong vascular differentiation, which clinically manifests as granuloma. According to the results of the cytochemistry, LCH was positive for smooth muscle actin while NLCH was negative [7].

Delving into the histological intricacies, Kuo Yuan et al. provided illuminating insights through their study. Employing immunochemistry on various target proteins, including vascular endothelial growth factor (VEGF), basic fibroblast growth factor (bFGF), thrombospondin-1 (TSP-1), angiostatin, and estrogen receptor (ER), their investigation revealed a strikingly elevated proportion of VEGF-positive cells [8]. On the other hand, Joy Thomas Vara and colleagues unraveled a significant connection between vascular and inflammatory parameters in the emergence of pyogenic granuloma. This revealed an expression site for VEGF further substantiating the role of inflammatory proliferation. In the early stages of pyogenic granuloma, conspicuous indicators include inflammatory edema and a substantial infiltration of macrophages, potentially accompanied by neutrophils and a few lymphocytes. These elements underscore the critical involvement of inflammatory mediators in the genesis of pyogenic granuloma [9]. This multidimensional exploration enriches our understanding of the condition and highlights the intricate web of factors orchestrating its development. Next, it is important to consider the distinguishing features of peripheral ossifying fibroma and gingival fibroma. Peripheral ossifying fibroma is more commonly seen in individuals aged between 30 and 40 years and occurs equally in both males and females. It is typically found in the anterior maxilla. Clinically the surface of the lesion is usually intact, although ulceration may occasionally be observed. Most often peripheral ossifying fibromas have a sessile growth pattern. Radiological features are mainly evident in long-standing cases, with some showing signs of osteolysis while others may exhibit a periosteal bone reaction. Histologically, peripheral ossifying fibromas are characterized by the presence of numerous fibroblasts, which proliferate extensively and produce mineralized material. This mineralized material may include bone cementum-like material, dystrophic calcification, or a combination of these components. On the other hand, gingival fibroma is typically seen in individuals aged between 20 and 50 years with an equal distribution among males and females. It is most commonly encountered in the anterior mandible region. Clinically, gingival fibromas have a uniform color and can be palpated to reveal evidence of fibrous stroma. These lesions do not tend to bleed and often exhibit a pedunculated growth pattern. In long-standing cases, radiological examination may reveal cupping-type bone resorption of the alveolar crest bone. Histologically, gingival fibromas comprise a fibrous stroma densely packed with collagen fibers. Hence, understanding the clinical, radiological, and histological features of pyogenic granuloma, peripheral ossifying fibroma, and gingival fibroma is crucial for making an accurate differential diagnosis in patients presenting with similar oral lesions [10-12].

As for the root cause of pyogenic granuloma's development, it remains an enigma. The condition's association spans a spectrum of clinical contexts, prompting a multifaceted exploration. Numerous authors advocate multifactorial etiopathogenesis, given that studies have unveiled divergent findings. Some investigations posit pyogenic granuloma's emergence as a physiological reaction to insults. In contrast, others counter that it arises in response to secondary stimuli like trauma, local irritation, bacterial infections, and similar triggers. The spectrum of contributing factors proposed by Kerr [13] includes botryomycosis, staphylococci infections, the presence of foreign objects, and even the accumulation of conditions within blood vessel linings. This intricate tapestry of insights underscores the complex mosaic that drives the disease's progression. Bacterial staining unveiled an immediate presence of gram-positive and gram-negative bacteria within the lesion, as outlined by Bhaskar and Jacoway [14]. Furthermore, the insights from Agha-Hosseini et al. [15] underscore a clinical perspective wherein the gingiva tends to thicken during pregnancy and, conversely, undergo atrophy more frequently during menopause. Their observations offer an intriguing facet, suggesting the gingiva's susceptibility as another target organ for the direct influence of hormones, specifically estrogen and progesterone. This illumination into hormonal interplay augments our comprehension of the complex dynamics influencing this anatomical context.

According to the findings of Whitaker et al. [16], the count of progesterone and estrogen receptors does not wield control over the underlying pathophysiology of this condition. Instead, the escalation of circulating hormones assumes a pivotal role in this context. Notably, the phase of pregnancy triggers a significant surge in progesterone and estrogen levels, potentially wielding a more pronounced impact on the lesion's endothelium. This heightened hormonal influence accentuates our understanding of the intricate dynamics within the lesion's milieu. A parallel exploration by Ojanotko-Harri et al. [17] demonstrated that pregnancy has the intriguing capacity to hinder the migration of inflammatory cells and fibroblasts. Numerous investigations have affirmed that pregnancy orchestrates progesterone metabolism while directing the trajectory of proinflammatory cells within the tissue. This indirect implication emphasizes that when the synchronization between progesterone levels and the direction of inflammatory cells is perturbed, the stage is set for the emergence of pyogenic granuloma [18]. Assim Banjar et al., in their comprehensive case report, offered an insightful perspective. They proposed mal occluded teeth-induced trauma as an underlying etiology for pyogenic granuloma. Notably, their documentation spotlighted the recurrent trauma inflicted on the lower lip labial mucosa due to the upper central incisor, culminating in the manifestation of pyogenic granuloma [19].

In our treatment plan, we opted for surgical excision under local anesthesia. This approach has exhibited promising treatment outcomes with minimal examples of recurrence. Another pertinent case, documented by Sara Ghadimi et al., involved the utilization of a diode laser for lesion treatment [20]. The literature boasts a variety of reported options for treating pyogenic granuloma, including lasers of diverse wavelengths. Dental lasers, introduced over four decades ago, have revolutionized oral soft tissue procedures. They offer precise incisions, immediate coagulation, and minimal postoperative discomfort and swelling. Several laser types, including Nd:YAG, carbon dioxide, Er:YAG, and diode lasers, have effectively treated pyogenic granuloma. An illustrative case by Maha A. Al-Mohaya et al. from Saudi Arabia showcased the employment of a 940 nm diode laser for the excision of pyogenic granuloma in patients with type II diabetes mellitus. While yielding subtle outcomes with minimal procedural events, recurrence remained possible [21]. Surgical excision, without doubt, attains the most promising results. Recent exploration has even delved into using sclerotherapy for pyogenic granuloma treatment. Applying substances like timolol or propranolol and topical or oral beta-adrenergic receptor antagonists has shown potential improvement, particularly in treating young children [22]. The chosen modality for treatment plays a pivotal role in diminishing the likelihood of recurrence. Significantly, it has been unveiled that approximately 16% of cases experience recurrence due to the failure to address underlying secondary factors that could have contributed to pyogenic granuloma's inception. The literature underscores surgical excision as the preferred treatment of choice [23]. However, an uncommon case reported by E. Parisi et al. highlighted multiple recurrences, unveiling a histological presentation featuring satellite lesions that might underlie the recurrent nature. Consequently, in circumstances that arouse suspicion, a meticulous 1 cm clearance should be undertaken during excision, and the removal of the periosteum should be contemplated in instances exhibiting pronounced invasiveness [24]. Remarkably, our present case was meticulously followed up over six months, revealing no recurrence.

## Conclusions

To conclude, pyogenic granuloma is a common lesion found in both the skin and oral cavity, particularly affecting the gingiva. This case report delves into a specific instance of gingival pyogenic granuloma in a female patient, shedding light on various aspects, including causes, clinical features, histological characteristics, treatment options, and the potential for recurrence. Laser technology has gained prominence in dental surgery for its user-friendliness benefiting both the dentist and the patient. Laser procedures, like excising exophytic lesions, are among its applications. Laser energy heats, coagulates, denatures proteins, vaporizes, and carbonizes cells. While most laser wavelengths and equipment tend to be costly and bulky, making them less cost-effective for simple surgical excisions. On the other hand, conventional scalpel surgical excision of gingival lesions involves cutting down to the periosteum and thorough scaling of adjacent teeth to remove potential sources of ongoing irritation. Conservative surgical excision, often curative, remains the preferred choice for treating pyogenic granuloma. This case underscores the trajectory of diagnosis and treatment, highlighting the successful outcome achieved through meticulous excision with adequate margin and base and careful monitoring over six months during which no recurrence was observed. This comparison not only underscores the complexity of the condition but also highlights the effectiveness of the chosen approach in achieving a positive result.
